# Childhood Leukemia Clusters in Yemen: The Dual Impact of Environmental Carcinogens and Healthcare Collapse in Conflict Zones

**DOI:** 10.7759/cureus.82396

**Published:** 2025-04-16

**Authors:** Assil N AlQubati

**Affiliations:** 1 Pediatrics, Al-Kuwait Hospital, Sanaa, YEM

**Keywords:** cancer clusters, displacement, environmental carcinogens, healthcare access, healthcare collapse, leukemia, public health, war, war and health, yemen

## Abstract

Yemen’s escalating childhood leukemia crisis stems from war-induced environmental contamination and systemic healthcare collapse. Prolonged conflict has released toxic agents - including heavy metals and radioactive residues - into ecosystems, disproportionately harming children during critical developmental stages. Concurrent destruction of medical infrastructure has rendered nearly half of healthcare facilities non-functional, delaying diagnoses and restricting treatment access. Childhood leukemia cases reported by Yemen’s Ministry of Health surged fivefold during the conflict, with 30% of cancer patients being children. However, these figures likely underestimate the crisis due to systemic underreporting from destroyed laboratories, displacement, and diagnostic barriers in inaccessible regions. This mirrors patterns in conflict zones like Fallujah (38-fold leukemia increase) and Gaza (heavy metal accumulation in infants). Unlike these regions, Yemen lacks foundational epidemiological tools - such as population-based cancer registries or genetic toxicity assays - to delineate leukemia clusters and their causes. Without such data, contamination mapping and accountability remain unattainable. Displacement and poverty exacerbate late-stage diagnoses and mortality. Urgent action must prioritize, investigating leukemia clusters through epidemiological methodologies and biomonitoring, mapping contamination sources; and rebuilding oncology infrastructure while halting military actions that perpetuate toxicity and deepen healthcare collapse. Yemen’s children exemplify war’s dual legacy: unstudied carcinogens and systemic healthcare abandonment, demanding accountability through global health ethics and intervention.

## Editorial

Yemen’s “no war-no peace” limbo since the 2022 ceasefire masks a growing tragedy: a cancer epidemic born not only from war’s suspected toxins but from its systemic erosion of healthcare. From Gaza to Fallujah, conflict zones bear witness to war’s carcinogenic signature - heavy metals, radioactive debris, and mutagens that linger for decades, poisoning soil, water, and bodies. Ongoing U.S. and UK airstrikes under maritime security pretexts deepen this crisis, threatening similar toxic legacies and destabilizing the fragile remnants of medical infrastructure.

Yemen’s Ministry of Health reports 90,000 registered cancer patients at the National Cancer Center in Sana’a, with 40,000 diagnosed during the war period; 30% are children [1]. Childhood leukemia cases have surged from 300 to 1,700 [1], a fivefold increase starkly disproportionate to Yemen’s population of 33 million. These figures, though alarming, likely underestimate the crisis due to systemic underreporting. The question is unavoidable: *Is war itself a carcinogen?*

During one of my shifts at the Pediatric Leukemia Center in Sana’a, a guardian arrived with his four-year-old nephew from Saada, whose leukemia diagnosis compelled them to flee their village. “*Why? What happened?*” he asked. “Six families in our village now have cancer. We survived the bombs but not this.” That same day, two additional pediatric cases presented: a five-year-old girl from Al-Jawf and a two-month-old infant from Haijah, both diagnosed with leukemia. All three cases originated from Yemen’s northern border regions, areas subjected to sustained Saudi-led coalition bombardment. This recurring epidemiological pattern cannot be ignored. The guardian’s questions mirror those documented in Fallujah and Gaza, underscoring the urgent need for investigation into conflict-associated carcinogenesis.

Beyond cancer, the war’s toxic legacy extends to birth defects, stunting, and genetic mutations - all unstudied in Yemen. In Fallujah, Iraq, post-2004 invasion leukemia rates surged 38-fold (RR=38.5; 95% CI: 19.2-77; *p*<0.00000001) among those under 34, infant mortality quadrupled (80 deaths/1,000 births vs. 9.7 in Kuwait), and birth sex ratios skewed toward girls - a hallmark of genetic damage linked to suspected uranium munitions [2]. In Gaza, newborns exposed to military strikes carried arsenic and barium (Group 1 and 2A carcinogens, respectively, per IARC) in their hair for years post-attacks, quantified via inductively coupled plasma mass spectrometry (ICP/MS), with contamination worsening by toddlerhood as toxins accumulated in their environment [3]. Heavy metals like molybdenum - implicated in esophageal cancer via oxidative stress pathways - were found in infants whose stunting mirrored Yemen’s wasting children, suggesting shared pathways of toxicity and malnutrition [3]. Yet Yemen lacks even basic studies to confirm clusters, let alone hold perpetrators accountable.

While suspected carcinogenic munitions demand scrutiny, war’s toxicity lies not only in contaminating land but in dismantling healthcare. Cluster bombs, documented by groups like Mwatana for Human Rights [4], scatter heavy metals across farmland, replicating Gaza’s arsenic-laced soil and Fallujah’s uranium legacy [2,3]. Meanwhile, 46% of Yemen’s health facilities are partially or completely non-functional due to shortages of staff, funds, medicines, and equipment [5]. Patients trek for months to reach Sana’a’s overwhelmed centers, often arriving with late-stage cancers. Radiotherapy units ration sessions to avoid shutdowns; chemotherapy drugs are scarce due to blockades. Families sell land for treatments that vanish mid-protocol, spiraling into destitution.

Displacement magnifies the crisis: 4.5 million Yemenis are internally displaced, while over 17 million cannot afford sufficient food and 21.6 million require humanitarian assistance - most of them women and children [5]. Cancer patients are stranded in clinics-turned-shelters or remote areas. “Cancer displaced us, not war,” lamented a Saada guardian. Even free treatment is unaffordable due to travel costs and lost wages.

Meanwhile, Gaza’s mothers retained stable arsenic levels over 18 months - proof of environmental entrenchment [3] - and Fallujah’s families faced generational genetic havoc [2]. Yemen’s airstrikes and siege risk cementing a third front in this carcinogenic trifecta: contamination, collapse, and silence.

Urgent action demands three non-negotiable steps. First, investigate clusters in high-suspicion regions using methodologies proven in Fallujah (population-based cancer registries, genetic toxicity assays) and Gaza (ICP/MS hair biomarker analysis) to map war’s carcinogenic footprint. Second, lift the blockade choking chemotherapy imports and rebuild radiotherapy infrastructure under WHO supervision to bypass political bottlenecks. Third, halt airstrikes exacerbating displacement, healthcare collapse, and environmental contamination that transforms battlefields into incubators of cancer.

Yemen’s children are casualties of war’s duality: its suspected carcinogens and its systemic collapse of healthcare. Their bodies, like those in Gaza and Fallujah, bear war’s invisible scars. Under the Helsinki Declaration’s mandate to protect vulnerable populations, their suffering demands accountability for both environmental poisoning and healthcare denial.
